# Comprehensive regional study of ESBL *Escherichia coli*: genomic insights into antimicrobial resistance and inter-source dissemination of ESBL genes

**DOI:** 10.3389/fmicb.2025.1595652

**Published:** 2025-06-10

**Authors:** Lisa Di Marcantonio, Sofia Chiatamone Ranieri, Michela Toro, Alice Marchegiano, Francesca Cito, Nadia Sulli, Ilaria Del Matto, Valeria Di Lollo, Alessandra Alessiani, Giovanni Foschi, Ilenia Platone, Massimiliano Paoletti, Nicola D’Alterio, Giuliano Garofolo, Anna Janowicz

**Affiliations:** ^1^Istituto Zooprofilattico Sperimentale dell’Abruzzo e del Molise “G. Caporale”, Teramo, Italy; ^2^Operative Unit of Clinical Pathology and Microbiology, Department of Services, Teramo, Italy

**Keywords:** antimicrobial resistance, One Health, ESBL genes, *E. coli* ESBL, cgMLST

## Abstract

**Introduction:**

The global dissemination of extended-spectrum β-lactamase (ESBL)-producing *Escherichia coli* (*E. coli*) poses a significant public health challenge, particularly in regions with high antimicrobial resistance (AMR) occurrence. This study investigated the occurrence, genomic characteristics, and dissemination dynamics of ESBL-producing *E. coli* in Abruzzo, Italy, by analyzing 956 isolates from humans, livestock, wildlife, and food products.

**Methods:**

Phenotypic and genomic analyses were performed on the isolates to assess ESBL-*E. coli* occurrence and characteristics. Multilocus sequence typing (MLST) was used to identify sequence types (STs), and plasmid profiling alongside synteny analysis was conducted to investigate horizontal gene transfer and resistance gene integration. Spatial analysis was also carried out to identify hotspots of ESBL-positive isolates.

**Results:**

An overall ESBL-*E. coli* occurrence of 14.1% (135/956 samples) was found, with significant variation across hosts: companion animals exhibited the highest occurrence (16.2%), followed by livestock and food matrices (14.6%), and wildlife (7.0%). Spatial analysis identified a hotspot in northeastern Abruzzo, where ESBL-positive isolates were 5.34 times more likely to occur (*p* < 0.001). MLST identified 58 sequence types (STs), with ST131 dominating human isolates (12/19). In cattle, predominant sequence types were ST16565 (5 isolates) and ST540 (4 isolates); in poultry, ST43 (5 isolates), ST10 (4 isolates), and ST6215 (3 isolates) were most common; ST206 (8 isolates) was predominant in swine; and in dogs, ST10 (4 isolates) and ST3580 (3 isolates) were most prevalent. Genomic analysis revealed host-specific distributions of ESBL genes: *bla*_CTX-M-15_ predominated in humans and dogs, while *bla*_CTX-M-1_ was most common in pigs. Plasmid profiling revealed IncF and IncI plasmids as key vectors for horizontal gene transfer. Synteny analysis showed identical flanking regions of *bla*_CTX-M-1_ and *bla*_CTX-M-15_ across phylogenetically distant strains, suggesting chromosomal integration and stable maintenance of resistance genes.

**Discussion:**

These findings underscore the interconnectedness of human, animal, and environmental reservoirs in AMR dissemination. The high genetic diversity observed within farms and the detection of shared clusters across hosts emphasize the need for integrated One Health interventions, including reduced antibiotic use in livestock and enhanced surveillance of high-risk environments. This study provides critical insights into local AMR dynamics, offering a model for regional mitigation strategies.

## 1 Introduction

The emergence and dissemination of ESBL-producing *E. coli* are driven by a multifaceted interplay across human, animal, and environmental ecosystems. In human healthcare, antibiotic overuse—particularly of third-generation cephalosporins—and nosocomial transmission in hospitals contribute to their selection and spread (Zamudio et al., 2024). Meanwhile, intensive livestock production amplifies resistance through prophylactic and therapeutic antibiotic use, facilitating transmission via the food chain (Ghimpețeanu et al., 2022). Environmental contamination further sustains this cycle, with wastewater, agricultural runoff, and wildlife acting as conduits for resistant bacteria and mobile genetic elements (Sharma et al., 2025; Guenther et al., 2011). International travel, urbanization, and inadequate antimicrobial stewardship further accelerate the cross-border dissemination of high-risk clones such as *E. coli* ST131 (Biggel et al., 2023). These interconnected pathways emphasize the critical need for a “One Health” approach to effectively monitor and mitigate this global health threat (Lv et al., 2024; Thanner et al., 2016; Franklin et al., 2024).

In Italy, despite a reduction in *E. coli* resistance to third-generation cephalosporins from 29.5% (2017) to 23.8% (2021), the prevalence remains significantly higher than the EU/EEA average (13.8%), placing Italy among the countries with highest resistance rates (European Centre for Disease Prevention and Control and World Health Organization, Regional Office for Europe, 2023). Resistance to aminopenicillins also remains elevated (>58%), reflecting widespread multidrug resistance. These data highlight the need for strengthened antimicrobial stewardship, as national resistance levels surpass most European benchmarks (5.5–37.3% for cephalosporins in 2021).

Recent studies have demonstrated the establishment of ESBL-producing *E. coli* in diverse reservoirs, including wildlife, agricultural environments, food products, companion animals, and human clinical isolates (Quinteira et al., 2024; Liu et al., 2024; Guibert et al., 2025). Wildlife has emerged as an environmental sentinel, reflecting the presence of resistant bacteria in natural ecosystems (Larsson and Flach, 2022; Mousavinezhad et al., 2024). Wild animals are exposed to antimicrobial agents and resistant bacteria via contaminated water, agricultural runoff, and anthropogenic waste, potentially acting as vectors bridging natural and human-modified ecosystems (Pérez-Rodríguez and Taban, 2019; Laborda et al., 2022). Increasing overlap between wildlife habitats and agricultural or urban areas facilitates both bacterial dissemination and horizontal gene transfer across reservoirs (Dolejska and Papagiannitsis, 2018; Abbassi et al., 2022).

Similarly, livestock systems promote resistance selection through intensive antibiotic use as growth promoters, prophylactics, or therapeutics (Van Boeckel et al., 2017; Islam et al., 2024). Livestock environments provide ideal conditions for the persistence and transfer of resistance genes via plasmids, transposons, and integrons (Carattoli, 2013; Zheng et al., 2025). Food products of animal origin can serve as direct transmission routes to humans, amplifying public health risks (Seiffert et al., 2013; Kürekci et al., 2024). Companion animals also act as reservoirs and vectors of ESBL-producing *E. coli*, facilitating interspecies gene flow through close human-animal interactions (Rubin and Pitout, 2014; Vračar et al., 2023). The economic impact is substantial, with ESBL-related infections prolonging hospital stays and increasing treatment costs by 200–300%, totaling €1.5 billion annually in the EU (Cassini et al., 2019; OECD, 2018).

Although significant progress has been made in molecular epidemiology, critical knowledge gaps persist regarding the drivers and pathways of ESBL-producing *E. coli* dissemination. Understanding interactions among wildlife, livestock, food products, companion animals, and humans remains essential for identifying selection pressures and mechanisms of resistance gene transfer (Mounsey et al., 2024; Holmes et al., 2016).

Against this backdrop, this study aims to assess the occurrence and molecular characteristics of ESBL-producing *E. coli* across diverse sources including wildlife, livestock, dairy products, and human clinical isolates in the Abruzzo region of Italy. By integrating genomic data, the study seeks to elucidate the distribution of ESBL genes and mobile genetic elements across hosts, contributing to a broader understanding of AMR dynamics within a “One Health” framework and informing targeted mitigation strategies.

## 2 Materials and methods

### 2.1 Sampling and statistics

This study investigated the occurrence, phenotype, and genotype of ESBL-producing *Escherichia coli* isolates collected between January and August 2023. A total of 956 samples were analyzed, covering multiple sectors and ecological contexts. All sample collections were conducted exclusively in 2023, following harmonized procedures for traceability and reproducibility. Detailed descriptions of sample sources, collection methods, and locations are provided below.

Samples from Livestock and food production (n = 519) were collected from breeding farms, slaughterhouses, food processing plants, and retail establishments handling food of animal origin. The farm-level sampling included broiler chickens, fattening pigs, sheep, and beef cattle. At each site, environmental and animal samples (including feces, rectal swabs, and intestinal content) were collected by trained veterinary staff using sterile tools and following national biosafety guidelines. Slaughterhouse sampling targeted carcasses and surfaces in contact with animal products. Processing plant and retail samples focused on raw food items of animal origin, such as meat cuts and offal. These activities were coordinated with regional veterinary services under official monitoring programs.

Samples from wildlife (n = 301) were collected during regional passive surveillance activities for African Swine Fever (ASF) and Chronic Wasting Disease (CWD). The sampling was conducted by authorized wildlife officers and veterinarians across multiple sites in the Abruzzo and Molise regions. Animal carcasses found dead or killed in road accidents were examined. Samples included intestinal contents, fresh feces, and internal organ tissues. All samples were geo-referenced using handheld GPS devices and coded for traceability.

Samples were collected from companion animals, domestic dogs (n = 99) and cats (n = 18) admitted to veterinary clinics for diagnostic or preventive purposes. Rectal swabs and fecal samples were collected during routine examinations or upon hospitalization, always with informed consent from the pet owners. For one cat, a brain tissue sample was included, collected post-mortem for unrelated diagnostic purposes.

Human clinical *E. coli* isolates were obtained from blood and urine samples of 19 patients in a local hospital in the same geographic area. These samples were provided by the hospital’s microbiology laboratory under ethical approval, and all isolates had been previously confirmed as ESBL-positive through standard diagnostic workflows. As no information was available about the total number of tested patients or ESBL-negative isolates, human samples were excluded from statistical comparisons.

Proportions were calculated with exact binomial 95% confidence intervals (Clopper-Pearson method). Group comparisons used Pearson’s χ^2^ test with post-hoc Fisher’s exact tests (Bonferroni-adjusted for multiple comparisons). Trend analysis employed the Cochran-Armitage test. Effect sizes included odds ratios (Baptista-Pike 95% CIs) and absolute risk differences. The Number Needed to Sample (NNS) was derived as the reciprocal of risk differences. All analyses were performed in R (v4.3.1) with *α* = 0.05. Additional methodological details are provided in Supplementary material 1.

### 2.2 Sample processing

Samples were collected using standardized protocols to ensure representativeness. All samples were transported in temperature-controlled containers at 4 ± 2°C and delivered to the laboratory within 2 h. Fecal samples (1 g ± 0.1 g) were homogenized in 9 ml of sterile buffered peptone water (BPW), while food samples (25 g ± 0.1 g) were homogenized in 225 ml of BPW.

Samples collected in buffered peptone water (BPW; Oxoid, United Kingdom) were incubated under aerobic conditions at 37 ± 1°C for 24 h. Subsequently, 10 μl of each enriched broth were plated onto MacConkey agar supplemented with 4 μg/ml cefotaxime (Sigma-Aldrich, Germany) to select for cefotaxime-resistant *Escherichia coli*. The inoculated plates were then incubated aerobically at 37 ± 1°C for 18 to 22 h.

Subsequently, 10 μl of each enriched broth were plated onto MacConkey agar supplemented with 4 μg/ml cefotaxime (Sigma-Aldrich, Germany) to select for cefotaxime-resistant *Escherichia coli*. The inoculated plates were then incubated aerobically at 37 ± 1°C for 18 to 22 h. Up to three presumptive *E. coli* colonies were subcultured onto fresh MacConkey Agar plates (4 μg/ml cefotaxime) and incubated as above. Colonies demonstrating consistent growth were considered potential ESBL-producing *E. coli*, confirmed colonies were stored in Microbank™ at −80°C.

Species identification was performed using the MALDI Biotyper system (Bruker Daltonics, Billerica, MA, USA) with the MALDI Biotyper Compass software version 4.1.

### 2.3 Screening for ESBL-producing strains and statistics

Antimicrobial susceptibility testing (AST) was performed on *E. coli* isolates grown on McConkey agar supplemented with cefotaxime to confirm ESBL production. Antimicrobial susceptibility testing (AST) was performed using the disk diffusion method in accordance with current EUCAST guidelines (EUCAST, s.d.). Epidemiological cut-off values (ECOFFs) established by EUCAST were applied to interpret the results in order to capture the presence of acquired resistance mechanisms such as ESBLs, as phenotypic breakpoints for some strains may not reflect resistance accurately in non-clinical isolates. Briefly, 0.5 McFarland suspensions of *E. coli* were inoculated onto Mueller-Hinton agar plates (Bio-Rad, France). Antibiotic disks were applied, and plates were incubated at 35 ± 1°C for 18 ± 2 h in aerobic conditions (Åhman et al., 2022). ESBL production was assessed using a combined disk diffusion test with cefotaxime/clavulanic acid (30 μg + 10 μg) and ceftazidime/clavulanic acid (30 μg + 10 μg) disks (ESBL disc kit, Liofilchem®, Roseto degli Abruzzi, Italy). *E. coli* ATCC 25922 and *K. pneumoniae* ATCC 700603 served as negative and positive controls, respectively. Inhibition zone diameters were measured, and an increase of ≥5 mm in the zone around the combination disks (ceftazidime/clavulanic acid or cefotaxime/clavulanic acid) compared to the corresponding single-antibiotic disks (ceftazidime or cefotaxime) was interpreted as ESBL production (Fröding et al., 2016).

ESBL-positive proportions were calculated with 95% confidence intervals using the Clopper-Pearson exact method. Between-group comparisons were performed using the Pearson chi-square test, followed by pairwise comparisons using the Fisher exact test and Bonferroni correction for multiple comparisons. Trend analysis was performed using the Cochran-Armitage test. Effect sizes were expressed as odds ratios (with 95% confidence intervals calculated according to the Baptista-Pike method) and absolute risk differences. The NNS was derived as the inverse of the risk difference. Logistic regression analysis was also performed to assess the association between different groups and ESBL positivity. All analyses were performed using R (v4.3.1) with a significance level of *α* = 0.05. Further methodological details are available in Supplementary material 1.

The samples were analyzed using R per statistical tests, including the Chi-square test to determine any differences between categories, the Pearson correlation test to analyze the relationship between the number of samples and the percentage of ESBL-positive, and the Z test to compare the categories. Human samples were excluded from the statistical analysis. This is because the human samples provided by the source had already tested positive for ESBL, without any information on the number of ESBL-negative samples. As a result, the percentage of negative samples in the human population could not be calculated, making it difficult to compare the human data with those of other categories.

Spatial analysis of samples was performed using QGIS and SaTScan to identify significant clusters of ESBL resistance. Data on cases, controls, and geographical coordinates were entered into SaTScan to perform spatial analysis and identify potential spatial clusters. The Bernoulli model in SaTScan was selected to detect clusters by comparing the observed and expected distributions of binary case–control data within scanning windows, identifying areas with a statistically significant excess of cases based on the Bernoulli probability model (Supplementary material 1).

### 2.4 Whole-genome sequencing

A subset of 120 *E. coli* isolates was selected from among those that tested positive for ESBL production, using a stratified approach based on sample type (animal, food, human), geographic origin, and matrix. This strategy was adopted to ensure representative coverage of sources and to characterize the genetic diversity of resistance among ESBL-producing strains. Total genomic DNA was extracted from 120 *Escherichia coli* ESBL positive isolates using the Maxwell 16 Tissue DNA Purification Kit, following the standard protocol provided by the manufacturer. The concentration of the extracted DNA was measured using the Qubit DNA HS Assay (Thermo Fisher Scientific Inc., Waltham, MA, USA). Genomic DNA was sequenced using the Illumina NextSeq500 platform. Briefly, sequencing libraries were prepared using the Nextera XT kit (Illumina, San Diego, CA, USA) according to the manufacturer’s instructions and subsequently sequenced in paired-end mode (150 bp) using the NextSeq500/550 Mid Output v2 reagent cartridge. After demultiplexing and adapter removal, the quality of the reads was assessed using FastQC v0.11.5 (Andrews, 2010). Raw reads were further processed using Trimmomatic v0.36 (Bolger et al., 2014) with the following parameters: Leading: 25; Trailing: 25; Slidingwindow: 20:25. Genome scaffolds were assembled using SPAdes v3.11.1 with the following parameters: –k 21, 33, 55, 77; −careful (Bankevich et al., 2012). The quality of the assembled scaffolds was evaluated with QUAST v4.3 (Gurevich et al., 2013). The set of paired-end genome sequencing reads from *E. coli* obtained in this study was deposited in the Sequence Read Archive (SRA) and associated with Bioproject PRJNA1218927.

### 2.5 Bioinformatics analysis

Genomic DNA from 120 high-quality *E. coli* isolates was subjected to whole-genome sequencing and analyzed using several bioinformatics tools. Plasmid incompatibility (Inc) groups and beta-lactamase genes, including ESBL genes, were identified using ABRicate v1.0.1 with 100% coverage criteria and the PlasmidFinder (Carattoli et al., 2014), NCBI (Feldgarden et al., 2020), and ResFinder (Zankari et al., 2012) databases (all updated February 21, 2023). MOB-recon module from MOB-suite v3.0.0 (Robertson and Nash, 2018) was used to predict plasmidic or chromosomal localization of ESBL genes using default settings. Flankophile (Thorn et al., 2024) was employed to analyze the synteny of *bla*CTX-M-1 and *bla*CTX-M-15 by comparing their flanking regions. We utilized single linkage clustering, which groups isolates iteratively: an isolate joins an existing cluster if its allelic distance to any member already within that cluster is less than or equal to the defined threshold. The threshold of 10 alleles corresponds to the default setting for this task template in SeqSphere+. We explored the additional cut-offs of 5 and 20 alleles to evaluate the stability of the clustering results across different thresholds. cgMLST data was used to generate a minimum spanning tree (MST) and UPGMA tree, which were visualized using iTOL v7 (Letunic and Bork, 2019). Data visualization of antimicrobial resistance gene presence was performed using RAWGraphs (Mauri et al., 2017). Novel MLST sequence types were submitted to EnteroBase for designation.

## 3 Results

### 3.1 Regional occurrence and distribution of ESBL-producing *E. coli* across diverse hosts and environmental matrices

The analysis of 956 samples revealed the presence of ESBL-producing *E. coli* in 135 samples, corresponding to 14.12% of the total. The distribution of ESBL-producing *E. coli* varied among the host categories analyzed. In particular, the highest positivity rate was observed in companion animals, with 19 positive samples out of 117 tested (16.24%), followed by farm animals and environmental matrices with 76 positive samples (14.64%), and wildlife with 21 positive samples out of 301 (6.98%), according to antimicrobial susceptibility testing (AST) results (Supplementary material 1; Table 1).

**Table 1:** Distribution of samples and frequency of ESBL-producing bacteria in the different categories analyzed.

Category	Total sample	ESBL-positive samples (%)	Confidence interval (95%)
Wild animals	301	21 (6.98%)	4.38–10.41%
Environmental, animal and food matrices	519	76 (14.64%)	11.69–18.05%
Companion animals	117	19 (16.24%)	10.08–24.18%
Total	937	116 (12.38%)	

The table shows the total number of samples, the number of ESBL-positive samples, the percentage of ESBL-positive samples and the 95% confidence intervals for each category (wild animals, environmental and food matrices, and companion animals).

The ESBL positivity among the categories, the percentage of positives and the 95% confidence intervals are as follows: among wild animals the percentage is 6.98%, with a confidence interval between 4.38 and 10.41%; for environmental and food matrices is 14.64%, with a confidence interval between 11.69 and 18.05%; finally, for companion animals, the positivity of ESBL is 16.24%, with a confidence interval ranging from 10.08 to 24.18%.

Statistical analysis shows significant differences between some of the categories compared.

Among non-human sample, the occurrence of ESBL-producing *E. coli* was significantly higher in environmental and food production samples (14.6%) and in companion animals (16.2%) compared to wildlife (7.0%). On pairwise comparison, environmental samples were significantly more positive than in wild animals (absolute risk difference: −7.66 percentage points; *p = 0.0008*; OR = 0.44, 95% CI: 0.26–0.73), with NNS of 13. Companion animals were also significantly more positive than in wildlife (risk difference: −9.26%; *p* = 0.0027; OR = 0.39, 95% CI: 0.20–0.75), with an NNS of 11. No significant difference was found between environmental and companion animals (*p = 0.67*; OR = 0.88; RD = −1.60%).

A significant increasing trend in ESBL positivity was detected along the gradient from wild animals to environmental samples to companion animals, consistent with progressive exposure to selective pressures or anthropogenic influence (Cochran-Armitage test, Z = 3.24, *p* = 0.0012).

Finally, logistic regression also confirmed that both companion animal (OR = 2.36, 95% CI: 1.30–4.31, *p = 0.005*) and environmental samples (OR = 2.10, 95% CI: 1.25–3.55, *p* = 0.005) had higher odds of ESBL positivity relative to wildlife, once more in favor of the observed associations. (Supplementary material 1; Table 1).

**Figure 1:**
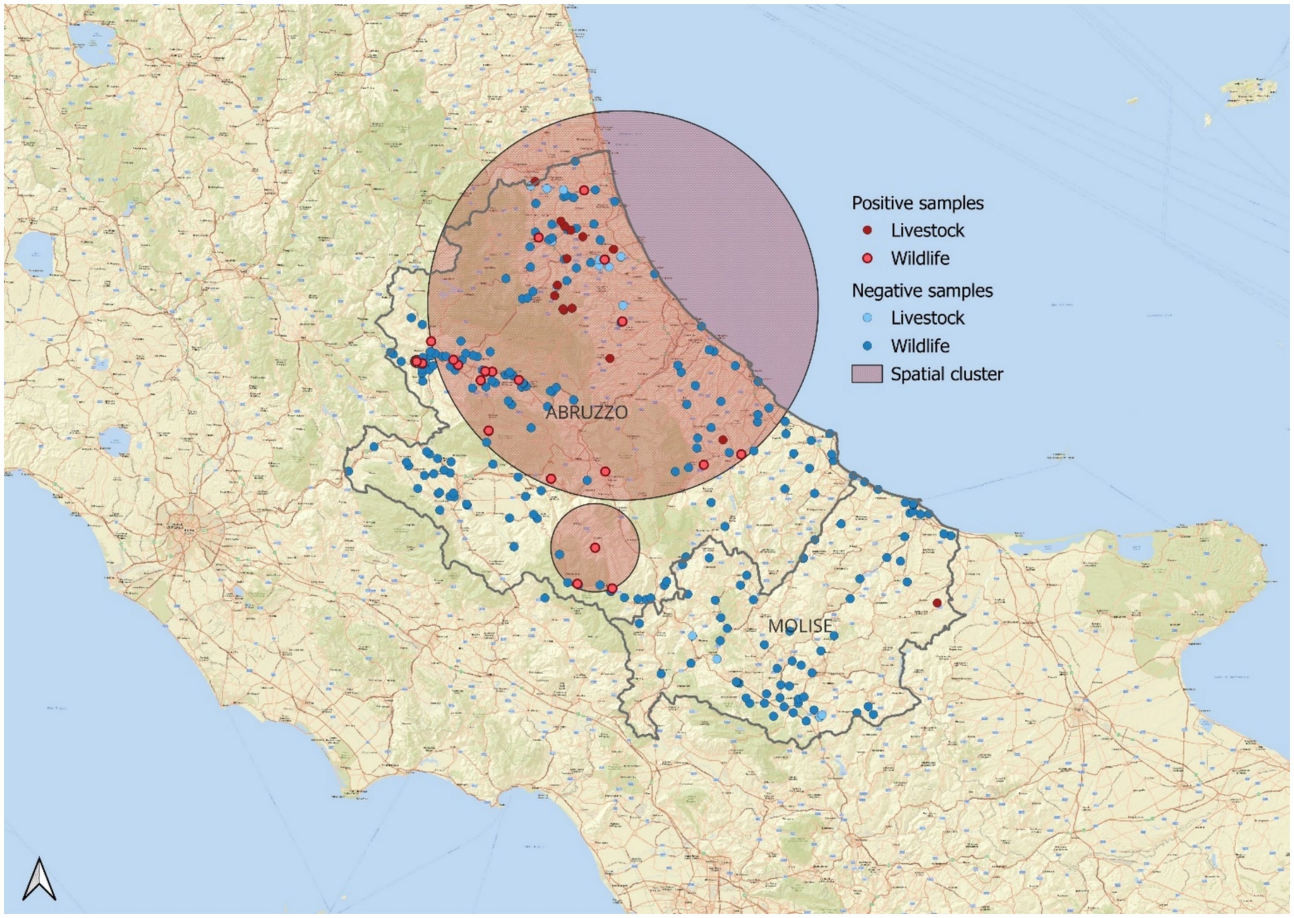
Statistically significant spatial cluster (*p* = 0.01) of ESBL-positive samples in wildlife and livestock populations. Positive samples are shown in red—dark red for livestock and lighter red for wildlife. Negative samples appear in blue—light blue for livestock and darker blue for wildlife. The relative risk (RR) is 5.34, indicating that the probability of detecting ESBL positivity within the highlighted area is 5.34 times higher than in the entire reference population. The observed-to-expected case ratio is 1.72, suggesting that the number of positive samples in this area is 1.7 times higher than expected.

SaTScan’s space-scan statistics identified spatial areas with observed occurrences that are higher than those expected by chance for Bernoulli-distributed variables. In particular, a cluster of ESBL-positive strains in the northeastern region was identified, with an observed to expected case ratio of 1.72. This cluster demonstrated a 5.34-fold increased probability of ESBL-producing *E. coli* isolation compared to the regional average (*p* < 0.001), indicating a significantly higher concentration of positive cases within this area (Supplementary material 1; Figure 1).

### 3.2 Genomic analysis reveals diverse sequence types with predominantly host-specific clustering of ESBL-producing *E. coli*

Whole-genome sequencing (WGS) was performed on 120 *E. coli* strains phenotypically confirmed as extended-spectrum ESBL producers. Multilocus sequence typing (MLST) identified 58 sequence types (STs), including three novel STs (Supplementary material 1). While most STs were represented by a single strain, some exhibited higher occurrence. Notably, ST-131 was the most frequent among human isolates (*n* = 12/19), ST-10 was frequently identified among isolates from dogs, poultry, and cattle (*n* = 10/64), although not exclusively dominant in each category. Additionally, ST-206 was identified in eight isolates from swine, representing the most frequent ST within that host group.

To further resolve the genetic relatedness among these isolates, core genome MLST (cgMLST) and cluster analysis were conducted (Supplementary Figure 1). Pairwise comparisons revealed a maximum allelic distance of 2,421 alleles, out of 2,513, examined between any two strains. Cluster analysis, employing allelic difference cut-offs of 5, 10, and 20 alleles, yielded similar numbers of clusters (16, 16, and 14, respectively), with the majority of clusters comprising only two strains. At the default threshold of 10 alleles, the largest cluster encompassed five strains isolated from diverse hosts, including two wild animals, two dogs, and one human, with pairwise allelic distances within this cluster ranging from 4 to 14. Interestingly, another cluster linked a human isolate with an isolate from a wild boar, separated by just 8 alleles. The remaining clusters were composed of strains originating from the same host species. Despite the predominance of ST-131 among human isolates, pairwise comparisons within this ST revealed substantial genetic diversity, with allelic distances generally exceeding 50 alleles. This high degree of genetic variation, even within the STs, underscores the significant diversity within the ESBL-producing *E. coli* population in Abruzzo. Among the sequence types identified in more than one host species, ST69 was found in human, wild boar, dog, porcupine, and deer isolates. While most ST were specific for a single isolation source, one of the most common STs, ST-10 contained strains from cattle, sheep and dogs. Within this ST we examined inter-source genomic diversity, which revealed considerable genetic distances between isolates from different hosts (maximum intra-ST distance: 992 alleles). The minimum pairwise cgMLST distance between ST-10 isolates from dogs and cattle was 294 alleles, whereas minimum distances involving poultry were substantially larger (562 alleles for dog-poultry; 591 alleles for poultry-cattle). Even higher maximum pairwise distances were observed between ESBL-producing *E. coli* strains isolated from different source types and assigned to different STs (>2,000 alleles).

To further explore the distribution of genetic variation, we examined the intra-host type diversity using cgMLST pairwise allelic differences (Supplementary material 3). Substantial genetic variation was evident within multiple host populations, with maximum pairwise differences exceeding 2,100 alleles in isolates from cattle, dogs, poultry, swine, wild boar, other wild animals, and sheep. Furthermore, multiple MLST Sequence Types (STs) were identified within most host groups, reinforcing the genetic heterogeneity observed within these populations. This finding demonstrates that the high overall genetic diversity of ESBL-producing *E. coli* observed in the Abruzzo region is not concentrated within a single host reservoir but is broadly distributed across the various animal sources investigated.

To investigate diversity at the farm level, we examined intra-farm cgMLST pairwise allelic differences (Supplementary material 3). Notably, substantial genetic diversity was present even within the confines of single farms. Several poultry, cattle, and swine farms (Farms 1, 3, 4, 8, 9) exhibited high maximum pairwise differences, often exceeding 2,300 alleles, indicating the co-circulation of highly divergent ESBL-producing *E. coli* lineages. This intra-farm diversity was also reflected in the detection of multiple MLST STs (up to 5 per farm). While some farms showed more limited diversity (e.g., Farm 2, Farm 7) or contained identical isolates (minimum distance of 0 on Farms 1, 2, 3, 5, 8), the overall data underscores that significant genetic heterogeneity can exist within individual farm environments in the Abruzzo region.

### 3.3 Phylogenetics and distribution of ESBL genes in *E. coli* isolates

**Figure 2:**
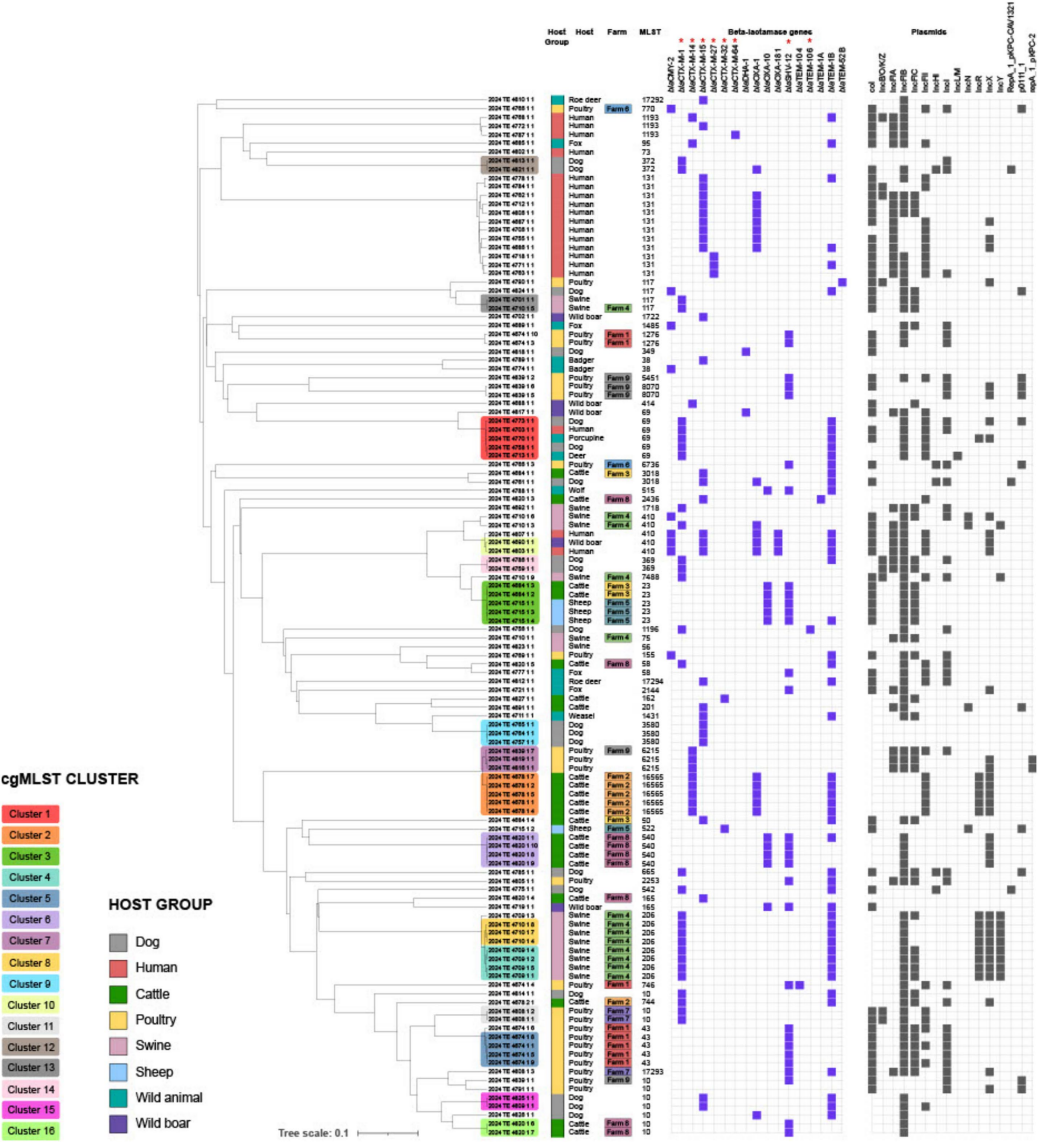
Dendrogram showing phylogenetic relationships between bacterial isolates from different hosts. Coloring in the “Host Group” column identifies the host group (e.g., dog, human, cattle, etc.). The middle columns report the specific host and the multilocus sequence type (MLST). Purple columns indicate the presence of β-lactamase resistance genes, while black columns represent isolate-associated plasmids. Genes classified as ESBL are specifically marked with an asterisk (*). The analysis highlights the spread of antibiotic resistance among different hosts and possible plasmid-mediated transmission.

**Figure 3:**
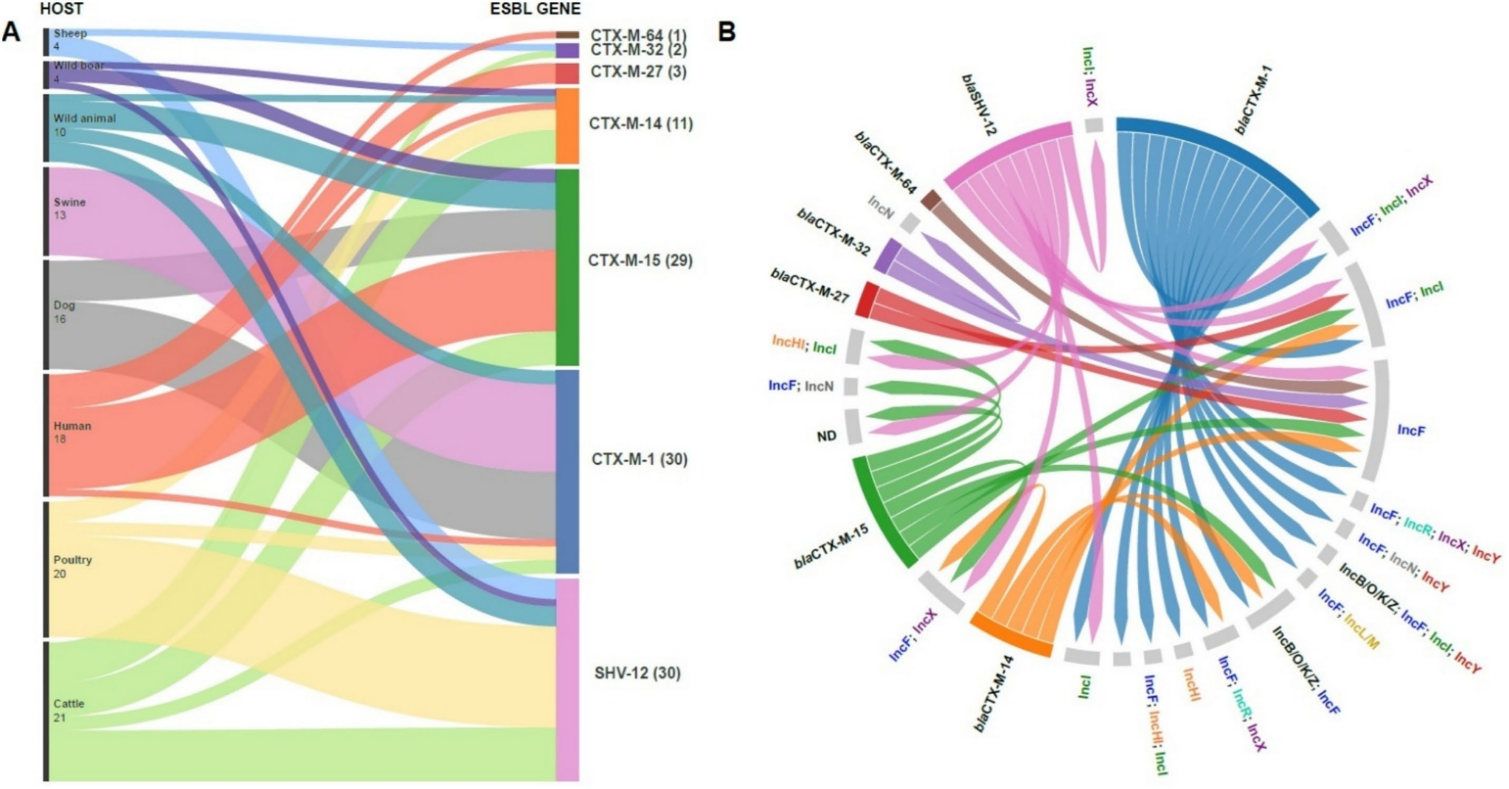
Distribution of antibiotic resistance genes among different host species. The Sankey diagram shows the flow of CTX-M and SHV-12 resistance genes among wild, domestic and human animals **(A)**. The number in parentheses indicates the number of isolates for each gene. The figure highlights the potential transmission of antibiotic resistance between animal and human environments, underlining the importance of monitoring antimicrobial resistance in public and veterinary health. Circular diagram representing associations between bla (extended-spectrum β-lactamase) antibiotic resistance genes and their host plasmids. Resistance genes (left) are linked to plasmids (right) by colored lines, indicating specific associations between genes and plasmid replicons. The presence of multiple connections suggests high genetic plasticity and horizontal transfer of resistance determinants between different plasmids **(B)**.

Analysis of *β*-lactamase genes, including ESBL genes and AmpC enzymes, revealed that most strains harbored multiple *bla* genes (Figure 2; Table 2), although ESBL genes were never found in duplicate unless in combination with AmpC genes. The majority of strains (76/120) carried ESBL genes belonging to the *bla*_CTX-M_ class, followed by *bla*_SHV-12_ (30/120). Within the *bla*_CTX-M_ class, the most prevalent variants were *bla*_CTX-M-1_ (30/120) and *bla*_CTX-M-15_ (29/120). Notably, *bla*_CTX-M-1_ was the dominant variant in swine isolates (13/15), whereas *bla*_CTX-M-15_ was frequently identified in human isolates (12/19). Additionally, *bla*_SHV-12_ was prevalent in poultry isolates (15/21). In wild animals, both *bla*_SHV_ and *bla*_CTX-M_ genes were detected, with *bla*_CTX-M-15_ predominating. Interestingly, specific ESBL gene variants were often found in strains isolated from the same host species (Figure 3A), even when those strains were phylogenetically distant, as demonstrated by cgMLST. This distribution of *bla* genes could therefore suggest potential host-specific adaptation and dissemination patterns of ESBL genes among *E. coli* strains in Abruzzo.

**Table 2:** Predominant sequence types, resistance genes, and plasmid incompatibility groups among ESBL-producing *Escherichia coli* isolates from different host categories.

Host category	Predominant sequence types	Most common resistance genes	Predominant plasmids
Human	ST131	*bla*_CTX-M-15_, *bla*_SHV-12_	IncF, IncI
Livestock	ST10, ST206, ST43, ST540, ST16565, ST23	*bla*_CTX-M-1_, *bla*_CTX-M-14_	IncI, IncX
Companion animals	ST3580, ST10	*bla*_CTX-M-15_, *bla*_CTX-M-1_	IncF, IncX
Wildlife	ST69, ST38	*bla*_CTX-M-1_, *bla*_CTX-M-14_	IncI, IncX

The table summarizes the most frequently identified sequence types (ST), the most common extended-spectrum β-lactamase (ESBL) genes, and the predominant plasmid groups associated with antimicrobial resistance in human, livestock, companion animal, and wildlife isolates.

### 3.4 Plasmid incompatibility group distribution and association with ESBL genes in *E. coli* isolates

Figure 3B illustrates the distribution of ESBL genes in strains carrying specific plasmid incompatibility (Inc) groups. The data reveal complex plasmid carriage, with individual isolates often harboring multiple plasmids or hybrid plasmids encompassing more than one Inc. group. ESBL genes frequently co-occurred with IncF plasmids, which were the most commonly identified plasmid type among our ESBL-producing *E. coli* isolates. All *bla*_CTX-M_ genes were associated with the presence of multiple Inc. groups, most commonly including IncF. *bla*_SHV-12_ showed a similar pattern, frequently found alongside IncF and IncI, often in combination. Several ESBL-producing strains lacked detectable Inc. groups. Due to the frequent assembly of ESBL genes, in particular *bla*_SHV-12_ and *bla*_CTX-M-14_, within short contigs using short-read sequencing, we were unable to reliably determine the specific plasmid localization of these genes. Table 3 shows the distribution of the main ESBL genes in the different plasmid replicons.

**Table 3:** Distribution of resistance genes, associated plasmids, and main host categories for ESBL-producing *Escherichia coli* isolates.

Resistance gene	Associated plasmids	Main hosts involved
*bla*_CTX-M-1_	IncF, IncI	Humans, Farm Animals
*bla*_CTX-M-14_	IncI, IncX	Wildlife, Farm Animals
*bla*_CTX-M-15_	IncF, IncX	Humans, Companion Animals
*bla*_CTX-M-27_	IncN, IncI	Wildlife, Farm Animals
*bla*_CTX-M-64_	IncHI, IncN	Wildlife, Farm Animals
*bla*_SHV-12_	IncN, IncX	Humans, Companion Animals
ND (not determined)	IncHI, IncI, IncF	Likely ubiquitous distribution

The table summarizes the most frequently detected ESBL genes, their associated plasmid incompatibility groups, and the main host categories in which these genes were identified.

### 3.5 Synteny analysis of *blaCTX-M* genes reveals potential horizontal gene transfer and chromosomal integration

**Figure 4:**
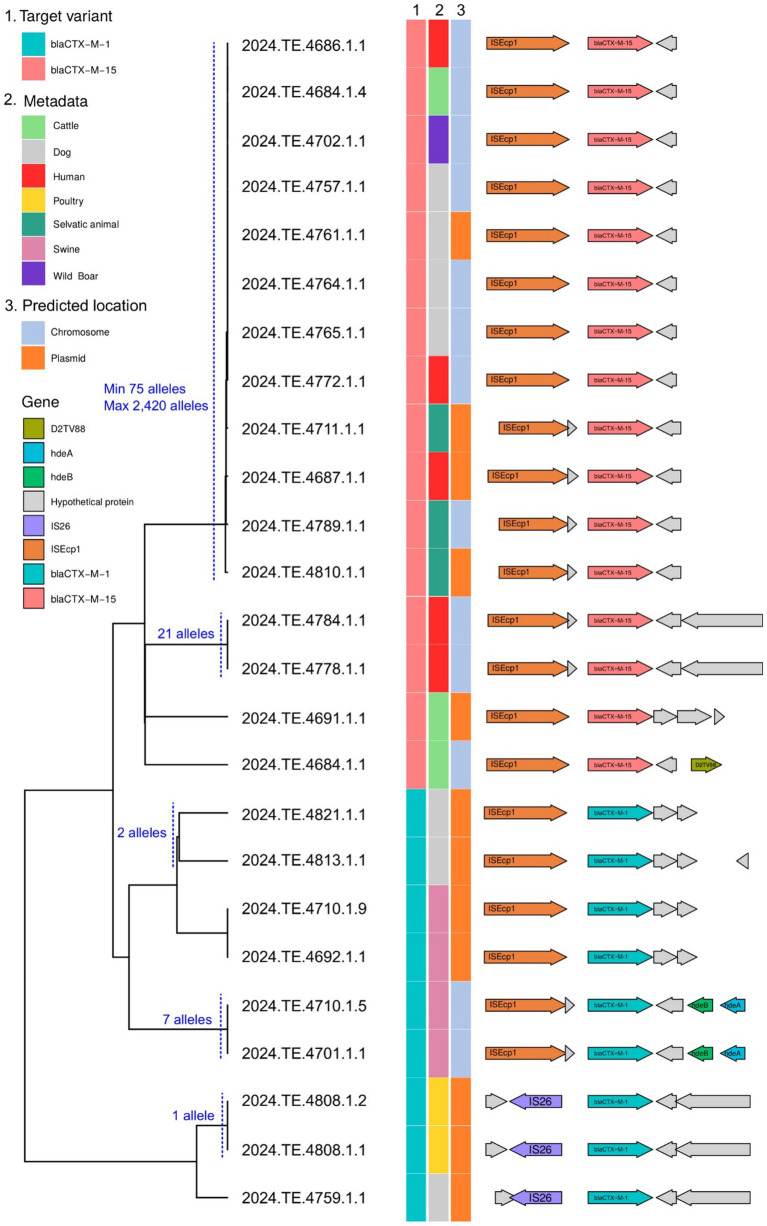
Dendrogram showing the phylogenetic relationships between sequences carrying the blaCTX-M-1 and blaCTX-M-15 resistance genes, isolated from different hosts (column 2). Column 3 indicates the predicted genomic location (chromosome or plasmid), as determined by the MOB-recon module of MOB-suite. On the right, the gene structure of the analyzed sequences illustrates the presence of mobile genetic elements, transposons, and other accessory genes associated with the dissemination of resistance. The cgMLST pairwise distance of 100 alleles or less between the strains is indicated. The combined analysis highlights the potential for interspecies transmission of resistance, facilitated by the mobility and plasticity of plasmids.

For ESBL genes located on longer contigs with at least 1,500 bp of flanking sequence, we analyzed gene synteny using Flankophile. This analysis included *bla*_CTX-M-1_ from 9 *E. coli* strains and *bla*_CTX-M-15_ from 15 *E. coli* strains. A distance tree based on flanking region sequences (Figure 4) revealed three main clades: two corresponding to *bla*_CTX-M-1_ and one to *bla*_CTX-M-15_. These clades were further subdivided into branches or smaller clusters, often containing strains from the same host. Notably, we identified a *bla*_CTX-M-15_ cluster containing strains from diverse hosts (e.g., dog, wild boar, human, and cattle) exhibiting identical gene synteny despite significant genomic divergence by cgMLST. This suggests potential horizontal gene transfer of the ESBL genes via mobile elements rather than clonal expansion. Intriguingly, gene location prediction indicated that *bla*_CTX-M-15_ in six of the seven strains within this cluster was likely chromosomally located. These findings highlight the complex dynamics of ESBL gene dissemination, involving both clonal expansion and horizontal gene transfer, and suggests the potential for a stable chromosomal integration of these resistance genes.

## 4 Discussions

This study highlights a clear gradient in the detection rates of ESBL-producing *E. coli* across ecological compartments, with the lowest proportion found in wild animals (6.98%—21 samples), and significantly higher rates in environmental samples (14.64%—76 sample) and companion animals (16.24%—19 sample). These findings are consistent with previous reports suggesting that wildlife, generally less exposed to anthropogenic antimicrobials, tends to harbor lower levels of resistance (Carroll et al., 2015). The occurrence data observed in our study are broadly consistent with previous reports. The detection rate of ESBL-producing *E. coli* in wild animals (6.98%) is comparable to the 5–10% range reported by Carroll et al. (2015) for European wildlife. Environmental samples showed an occurrence of 14.64%, which aligns with recent findings from the European Food Safety Authority and European Centre for Disease Prevention and Control (2024). ESBL-producing *E. coli* in companion animals (17.1% in dogs) was notably higher than the 6–12% range usually reported in Italian clinical surveys (Giufrè et al., 2021), indicating potential differences in exposure or antimicrobial stewardship practices. Livestock-associated prevalences observed in cattle and pigs were in line with those previously documented by Homeier-Bachmann et al. (2022), confirming the role of intensive farming in sustaining ESBL reservoirs.

The identification of a statistically significant geographic cluster of ESBL-producing isolates in northeastern Abruzzo highlights potential regional factors influencing resistance. This cluster suggests that environmental and anthropogenic elements such as agricultural runoff and wastewater contamination may contribute to the higher-than-expected occurrence of ESBL-positive samples in this area. It is important to note that the observed cluster does not necessarily represent an outbreak but indicates areas where resistance is more present. This geographic concentration underscores the need for further investigation into the environmental and social factors that could explain the spatial distribution of resistance. Targeted surveillance in these areas may help identify potential reservoirs and guide future control measures. The observed 2.3-fold increase from wild to companion animals suggests a strong influence of human-related antimicrobial use and environmental contamination, supporting the hypothesis of “resistance pollution” driven by human activity (Larsson and Flach, 2022).

Statistical analyses show significant differences in the occurrence of ESBL-producing *E. coli* between the different compartments examined. In particular, the lower frequency in wild animals samples compared to those from livestock and pets suggests a non-homogeneous distribution of the phenomenon, probably linked to the degree of exposure to selective sources such as antibiotics and anthropized environments. The comparison between wild animals and sample from environmental, animal and food matrices showed a significant difference, with an absolute risk lower by about 8 percentage points in wild animals. Samples from companion animals also showed a significantly higher values than wild animals (−9.26%; OR = 0.39), indicating a greater selective pressure, probably linked to the therapeutic use of antibiotics in the domestic environment. The lack of significant differences between environmental, animal and food matrices and pet samples could reflect a similar level of exposure to sources of resistance, both direct (antibiotic treatments) and indirect (environmental contamination). The trend analysis confirms the presence of an increasing gradient of positivity from the wild to the domestic compartment, consistent with a progressive intensification of contact with human or anthropized environments. This interpretation is further strengthened by the logistic regression model, which identifies a significantly higher probability of positivity in environmental, animal and food matrices samples compared to wild animals, also taking into account the sampling variability. These results underline the value of wild animals as a possible sentinel indicator of environmental background in areas with low anthropic pressure, and reiterate the need for integrated One Health approaches for the surveillance and containment of antibiotic resistance along the human-animal-environment continuum.

The significant increasing trend observed across the wild, environment animals and food matrices, companion animal continuum (Z = 3.24, *p =* 0.0012) reinforces the notion of a directional flow of resistance determinants, potentially driven by selective pressures across interfaces shaped by human impact. Moreover, the calculated NNS 13 and 11 for wild animals vs. environmental, animal and food matrices and wild animals vs. companion animals comparisons, respectively suggests that focused surveillance in these interfaces may allow for early detection of emerging resistance hotspots. Our environmental detection rate aligns with recent European food-chain surveillance data (European Food Safety Authority and European Centre for Disease Prevention and Control, 2025). However, the positivity rate among companion animals exceeds Italian clinical reference levels (typically 6–12%) possibly reflecting gaps in antimicrobial stewardship in veterinary settings. Given the close contact between humans and pets, these findings raise concerns about the potential for bidirectional transmission of resistant bacteria within household environments (Carattoli et al., 2005; Ratti et al., 2023).

Taken together, these results underscore the importance of integrated One Health surveillance systems that include wildlife, especially in transitional zones such as peri-urban and agricultural interfaces. Mediterranean ecosystems, characterized by dense human-wildlife-livestock interactions, represent critical settings where early signals of resistance amplification may emerge. Future studies should aim to include genomic characterization of isolates and assess environmental variables to better understand transmission pathways and resistance reservoirs. The identification of a statistically significant ESBL-*E. coli* hotspot in northeast Abruzzo suggests localized environmental or anthropogenic factors driving increased occurrence (Cocco et al., 2023). Potential contributors include agricultural runoff, contaminated water sources, and wildlife interactions, highlighting the interconnectedness of these ecosystems within a One Health framework (Leoni et al., 2023; Formenti et al., 2021). The spatial analysis conducted in this study identified an area in Abruzzo where the number of positive ESBL-producing *E. coli* samples exceeded the expected values based on the Bernoulli model. It is important to note that this analysis does not suggest the presence of an ESBL hotspot or outbreak, but rather highlights a region where the occurrence of positive samples is significantly higher than anticipated. This finding can serve as a basis for further targeted investigations to understand the underlying factors contributing to this elevated occurrence.

The red circular area shown in the map corresponds to the location of these positive samples, with a distinction made between domestic (livestock) and wildlife sources. However, this geographic analysis is not aimed at identifying a specific source of ESBL but rather to provide a preliminary insight into potential areas of interest for further surveillance. Future studies focusing on this region could help identify possible environmental, agricultural, or ecological factors that may be influencing the distribution of ESBL-producing bacteria.

The observed high occurrence of ESBL-*E. coli* in cattle and pigs reinforces the role of intensive farming practices in selecting for antimicrobial resistance, particularly through the use of third and fourth-generation cephalosporins. This aligns with existing literature indicating that prophylactic and therapeutic antibiotic use in livestock fosters the persistence and transmission of resistant strains to humans via contaminated meat (Homeier-Bachmann et al., 2022; Giufrè et al., 2021).

Management practices could influence the selection and spread of certain resistance genes. For example, the use of antibiotics in veterinary medicine could favor the selection of resistant strains with specific ESBL variants, such as *bla*_CTX-M-1_ in pigs (Homeier-Bachmann et al., 2022). This observation highlights the importance of targeted monitoring and control strategies, which take into account the differences between host species and their environments, to effectively counteract the spread of antibiotic resistance.

While wildlife exhibited lower ESBL-*E. coli* occurrence compared to farmed animals, its presence in wild boar, foxes, and badgers implies environmental exposure (Homeier-Bachmann et al., 2022; Formenti et al., 2021). Contamination of water and food resources by urban waste, livestock runoff, and hospital effluents likely facilitates the spread of resistant strains (Leoni et al., 2023). The persistence of ESBL-*E. coli* in the environment, coupled with its transmission through contaminated water, underscores the environmental dimension of AMR dissemination (Hong et al., 2020).

The 17.1% ESBL-*E. coli* occurrence observed in dogs, compared to the absence of positive samples in cats, may be due to a number of factors. Dogs are generally more exposed to outdoor environments, have frequent contact with other animals and humans, and are more likely to be treated with antibiotics, particularly for skin, ear, and urinary infections. This incidence in dogs may derive from veterinary antibiotic use, contaminated pet food, or exposure to polluted urban environments (Kristianingtyas et al., 2021). Cats, by contrast, have less exposure to the outdoors and are generally treated with fewer antibiotics, potentially reducing their risk of infection and transmission of resistant bacteria (Johnson et al., 2022; Formenti et al., 2021). Additionally, the lower sociality of cats compared to dogs could limit the inter-individual transmission of resistant strains. This behavioral and management difference likely contributes to the observed disparity in ESBL-*E. coli* carriage between the two species. Furthermore, the established link between ESBL-*E. coli* carriage in dogs and their owners necessitates further investigation within this region (Schmitt et al., 2021; Johnson et al., 2022).

The genomic analysis revealed a complex interplay of interspecies transmission and host-specific adaptation. While the close genomic relatedness of isolates from different hosts (e.g., human and wild boar) suggests potential zoonotic transmission or shared environmental reservoirs, the overall host-specific clustering indicates adaptation to particular host niches (Hong et al., 2020; Giufrè et al., 2021). It should be noted, however, that while strong host-specific clustering was observed for food animals (cattle, swine, poultry) and humans, this pattern appeared less distinct among wild animal isolates, possibly reflecting greater environmental exposure and less selective pressure compared to domesticated species. This is supported by the observed host-specific distribution of ESBL genes, with *bla*_CTX-M-1_ predominating in swine and *bla*_SHV-12_ in cattle. These observations are consistent with previous studies that reported the occurrence of *bla*_CTX-M_ genes in humans and animals, and specifically the high occurrence of *bla*_CTX-M-15_ in humans and cattle, and of *bla*_CTX-M-1_ in pigs (Hong et al., 2020). However, the geographic limitations of our study may have restricted our ability to capture the full diversity of circulating ESBL-*E. coli* strains. Likewise, the relatively small number of human clinical isolates analyzed (*n* = 19) represents a further limitation in terms of epidemiological representativeness. Nonetheless, these isolates were included not to estimate occurrence, but to offer a comparative genomic framework alongside strains from animal and environmental sources. Despite the limited sample size, they encompass key high-risk clones such as ST131, ST38, and ST69, which are globally disseminated and clinically relevant. Their presence in our dataset strengthens the evidence for cross-sector transmission and supports the added value of including human data in One Health genomic surveillance. In particular, the detection of clone ST69 in a wide range of hosts—including humans, wild boars, dogs, porcupines and deer reinforces the hypothesis of a broad environmental distribution and the potential for interspecific transmission of ESBL-producing strains. ST69 has been described as an emerging clone associated with urinary and systemic infections in humans, with an increasing capacity to spread also in non-hospital settings (Wang et al., 2024; Kuan et al., 2024). Its presence in wild and domestic species within the same geographical area suggests an ecological connectivity between anthropized and natural environments, placing ST69 as a further indicator of the need for integrated surveillance according to the One Health approach. Furthermore, the close genomic distance between some host specific clusters of isolates that harboured the same type of ESBL genes would suggest that the host-ESBL gene connection derives from the epidemiological relatedness of the isolates rather than the host type (Supplementary Figure 1; Figure 2).

The frequent occurrence of multiple plasmids, particularly IncF, and hybrid plasmids underscores their crucial role in ESBL gene dissemination. The co-localization of multiple resistance genes on single plasmids contributes to the complexity of AMR transmission (Schmitt et al., 2021). However, our use of short-read sequencing may have limited our ability to fully resolve the genomic context of ESBL genes, particularly in complex or repetitive regions. Long-read sequencing could provide more comprehensive insights into the genetic environment of resistance genes (Cocco et al., 2023).

Flankophile analysis revealed evidence of both host-specific co-evolution and horizontal gene transfer (HGT). The presence of the *bla*_CTX-M-15_ cluster with identical gene synteny across diverse hosts strongly supports HGT via mobile elements (Johnson et al., 2022). Furthermore, the likely chromosomal integration of *bla*_CTX-M-15_ in most strains within this cluster suggests a mechanism for stable resistance gene maintenance, even without constant antibiotic selection (Hong et al., 2020). This finding emphasizes the importance of considering both HGT and chromosomal integration in surveillance and intervention efforts aimed at controlling ESBL dissemination.

In conclusion, this study underscores the need for a One Health approach to combat ESBL-*E. coli* dissemination, recognizing the interconnectedness of human, animal, and environmental ecosystems. Future research should quantify the contributions of various transmission pathways, investigate host-specific adaptation mechanisms, and further characterize the roles of plasmids and chromosomal integration in resistance dissemination (Leoni et al., 2023). Longitudinal studies are essential to track the evolution and spread of resistance genes over time and to develop effective intervention strategies.

## 5 Conclusion

In summary, this research highlights the widespread diffusion of ESBL-producing *Escherichia coli* in human, animal and environmental compartments within a specific geographical region. The findings highlight the urgency of adopting integrated and collaborative strategies in the fight against antimicrobial resistance, emphasizing the need for rational use of antibiotics and continuous surveillance to safeguard human and animal health. In particular, the presence of these resistant bacteria in food products and livestock highlights the importance of enhancing monitoring throughout the food chain. To effectively address this challenge, a global, coordinated and targeted One Health approach is essential.

## Data Availability

The original contributions presented in the study are publicly available. This data can be found at: https://www.ncbi.nlm.nih.gov/bioproject/PRJNA1218927.
