# Supplementary figures and images for: Comprehensive regional study of ESBL Escherichia coli: genomic insights into antimicrobial resistance and inter-source dissemination of ESBL genes
Source: Front Microbiol. 2025 Jun 10;16:1595652. doi: 10.3389/fmicb.2025.1595652 (PMC12185426; doi:10.3389/fmicb.2025.1595652)

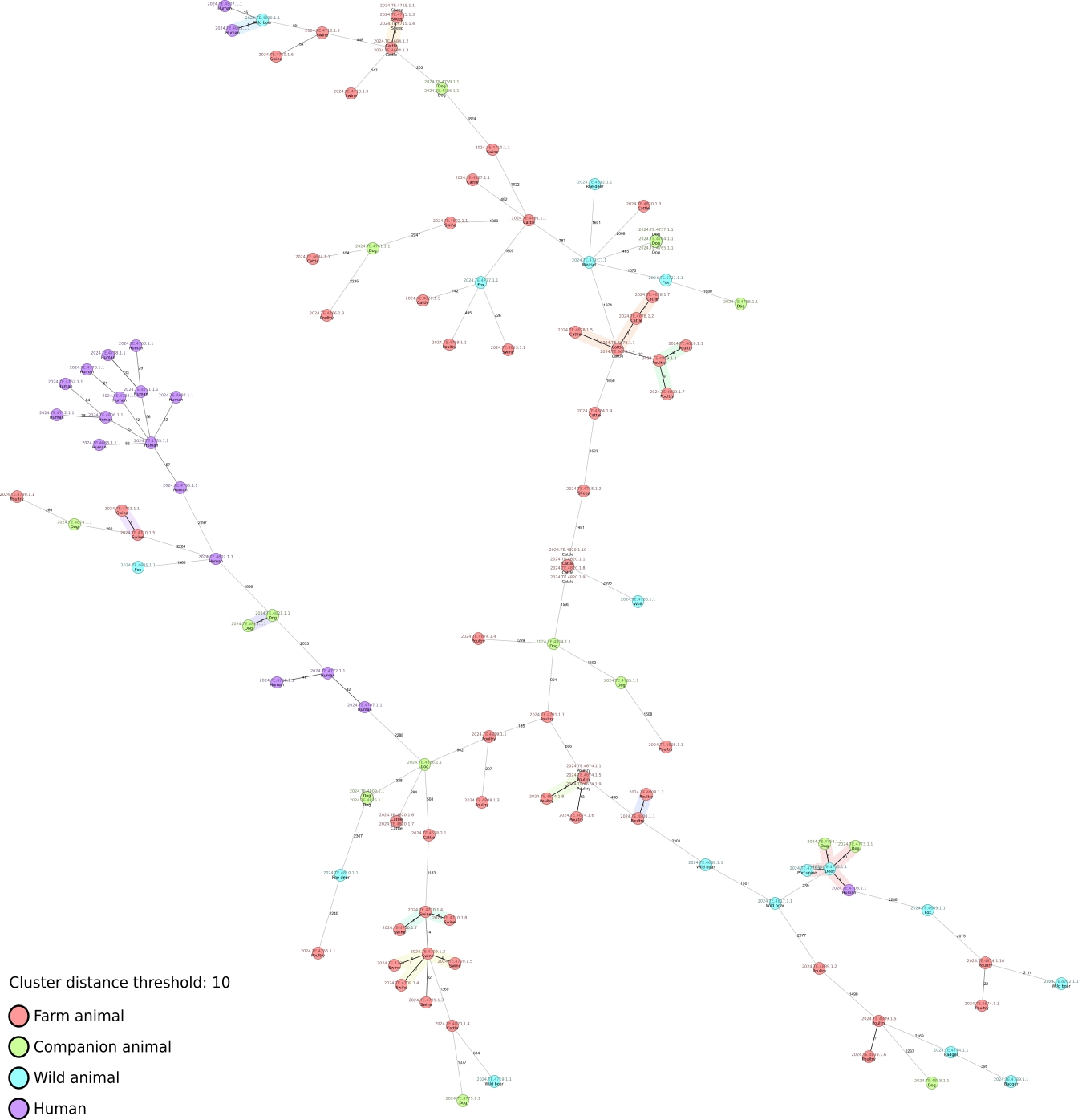

Supplement: Supplementary file 1 [file Image_1.JPEG]
